# Supplementary material for: Aerobic exercise training resets the human skeletal muscle methylome 10 years after breast cancer treatment and survival
Source: FASEB J. 2022 Dec 21;37(1):e22720. doi: 10.1096/fj.202201510RR (PMC13281840; doi:10.1096/fj.202201510RR)
Supplement: Supplementary file 1 — Figure S1 [file FSB2-37-e22720-s005.pdf]

1A

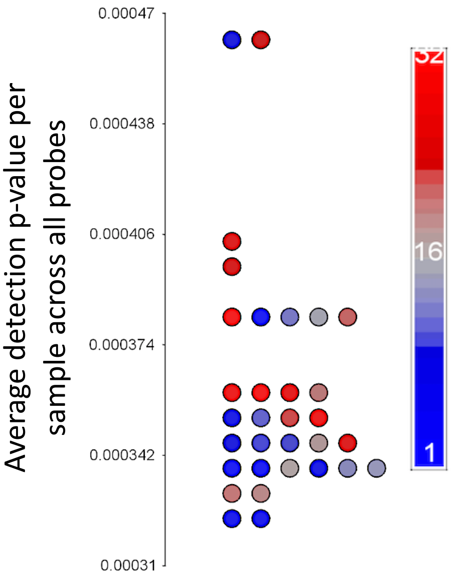

1B

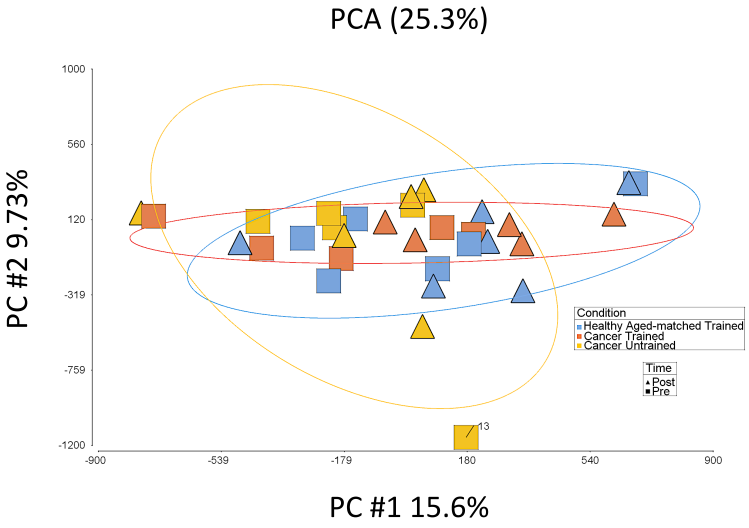

Suppl. Figure 1. A. Average detection p-value per sample across all probes, with all samples with values less than 0.0004. **B.** PCA for all individual samples across all conditions coloured by condition/group (Cancer Trained- orange, Cancer Untrained- yellow and Healthy Aged-matched Controls- blue) and shaped by time (pre and post training, squares and triangles respectively). One sample (sample 13) was removed due to a larger variation than that expected within that condition/group (defined as values above 2.2 standard deviations for that condition).
