# Supplementary material for: Aerobic exercise training resets the human skeletal muscle methylome 10 years after breast cancer treatment and survival
Source: FASEB J. 2022 Dec 21;37(1):e22720. doi: 10.1096/fj.202201510RR (PMC13281840; doi:10.1096/fj.202201510RR)
Supplement: Supplementary file 2 — Figure S2 [file FSB2-37-e22720-s004.pdf]

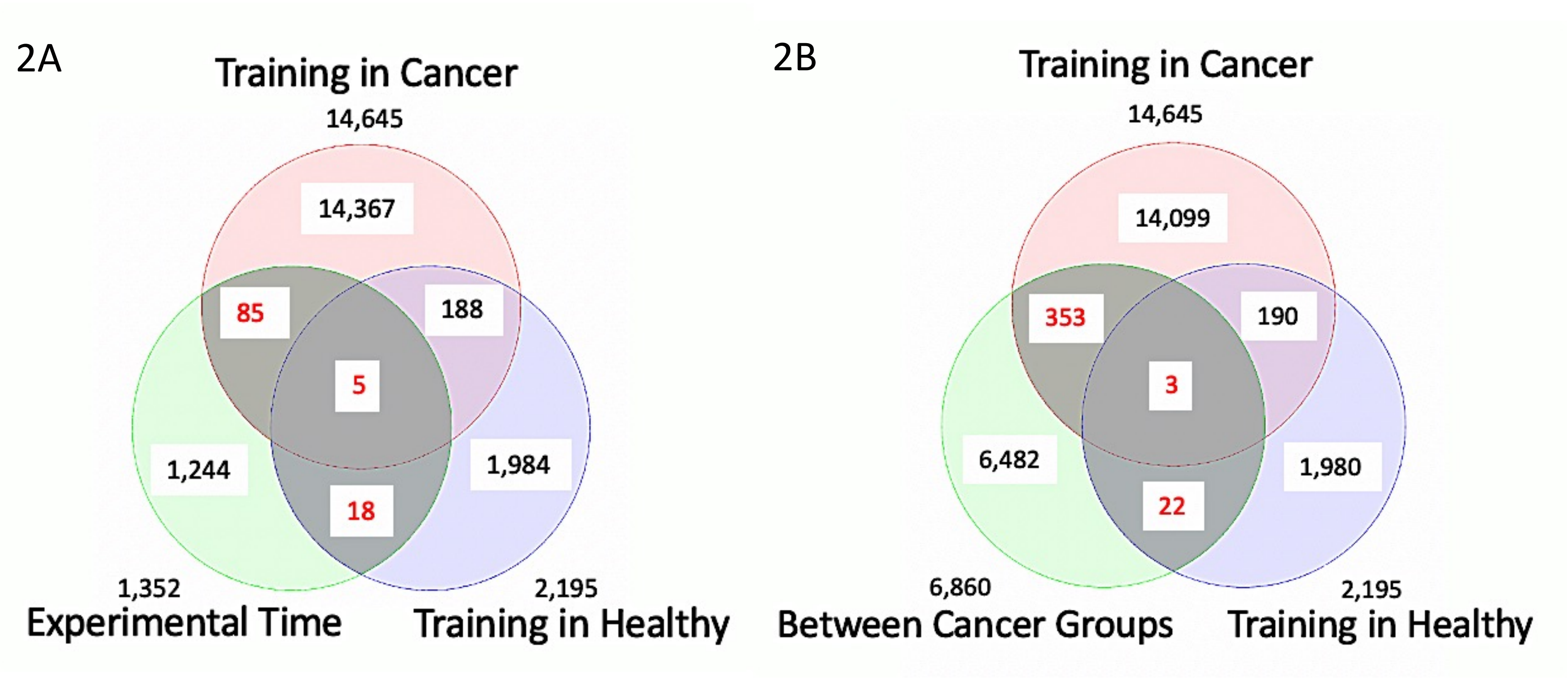

Suppl. Figure 2. (A) There were 1,325 differentially methylated positions (DMPs) that were altered over the experimental time of 5 months. Only 108 out of these 1,325 DMPs were influenced by aerobic training in either the cancer survivors or healthy age-matched controls (overlapping numbers in red font). These 108 DMPs were removed from later analysis so that any differential methylation observed to be altered with training was due to the training intervention and not a consequence of the experimental time period of the training intervention. **(B)** 6,860 DMPs identified between cancer survivor groups at baseline. Only 378 out of these 6,860 DMPs were influenced by aerobic training in both the cancer survivors and healthy age-matched controls (overlapping numbers in red font). Therefore, we also removed these 378 DMPs so that any downstream discovery of differential methylation would be due to the training intervention rather than having been influenced by starting differences in methylation between cancer survivor groups.
